# Landscape analysis of m6A modification regulators reveals LRPPRC as a key modulator in tubule cells for DKD: a multi-omics study

**DOI:** 10.3389/fphar.2025.1506896

**Published:** 2025-04-04

**Authors:** Li Jiang, Hongda Yu, Jie Jian, Xulin Sai, Yudian Wang, Yufei Zhang, Xiai Wu

**Affiliations:** ^1^ Diabetes Department of integrated Chinese and Western Medicine, China National Center for Integrated Traditional Chinese and Western Medicine, China-Japan Friendship Hospital, Beijing, China; ^2^ Dermatology Department, China-Japan Friendship Hospital, Beijing, China; ^3^ General Medicine Department, Mental Health Center of Dongcheng District, Beijing, China; ^4^ Dongzhimen Hospital Affiliated to Beijing University of Chinese Medicine, Beijing, China; ^5^ College of Traditional Chinese Medicine, Hubei University of Chinese Medicine, Hubei, Wuhan, China

**Keywords:** m6A modification, diabetic kidney disease, LRPPRC, tubules, single-cell transcriptome, spatial transcriptome

## Abstract

**Background:**

Diabetic Kidney Disease (DKD) is a serious complication of diabetes, imposing a substantial medical burden. The significance of N6-methyladenosine (m6A) modification in the pathogenesis of DKD has become increasingly prominent.

**Aim:**

This study aimed to investigate the specific expression patterns of the m6A geneset in the pathogenesis of DKD.

**Method:**

Bulk RNA, single-cell and spatial transcriptome were utilized to clarify the hub gene. 3 types of machine learning algorithms were applied. The possible compounds were screened based on the DSigDB database.

**Result:**

GSEA has revealed the potential m6a-associated pathways such as cGMP-PKG pathway. GSVA showed that the two types of m6a regulation, namely m6a-readers and m6a-writers, were generally suppressed in DKD patients. The output of 3 types of machine learning algorithm and differential analysis has determined the LRPPRC as the hub gene. LRPPRC was downregulated in the LOH, PODO, CT, and CD-ICB cell populations, most of which were tubular cells. It exhibited the decreasing trend over time, particularly pronounced in LOH cells. The low activity of LRPPRC was mainly detected in the injured renal tubules. In clinical patients, the expression levels of LRPPRC mRNA in DKD showed the tendency to be downregulated and exhibited the potential correlations with Glomerular Filtration Rate (GFR) and proteinuria according to the Nephroseq database. The lobeline might be an important potential compound involved in the regulation of LRPPRC and other m6a genes. Its actual efficacy needs to be verified *in vivo* or *in vitro*.

## Graphical Abstract

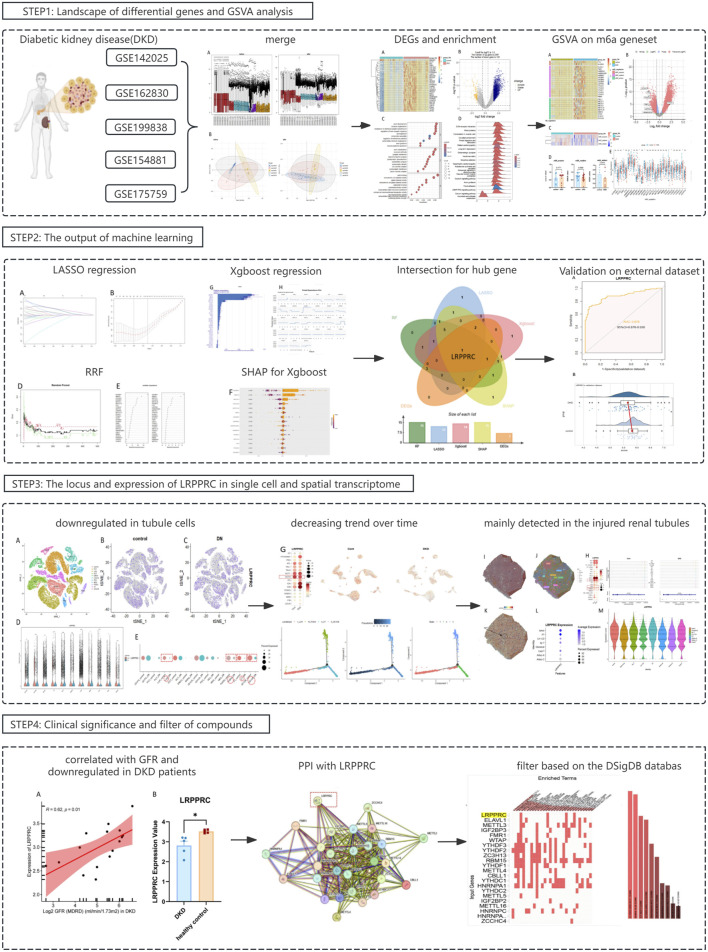

## 1 Introduction

Diabetic kidney disease (DKD), also known as diabetic nephropathy, is a serious complication of diabetes characterized by progressive kidney damage resulting from chronic hyperglycemia and associated metabolic disturbances. Early detection and management of DKD are crucial in preventing its progression and mitigating its associated health risks (Akhtar et al., 2020; Barutta et al., 2022).

In recent years, the pathogenesis of DKD has been deeply studied. The development and progression involve a complex interplay of various signaling pathways and cellular mechanisms.The TGF-β signaling pathway is a central driver of renal fibrosis, with TGF-β1 promoting epithelial-mesenchymal transition (EMT) in renal epithelial cells, leading to the loss of epithelial characteristics and acquisition of mesenchymal traits, ultimately contributing to fibrogenesis (Chatterjee et al., 2025). Similarly, Wnt signaling, through β-catenin activation, exacerbates renal fibrosis by inducing fibroblast activation and extracellular matrix (ECM) production, promoting the endothelial-mesenchymal transition (EndMT) (Hadpech and Thongboonkerd, 2024). Notch signaling, which regulates cell fate determination, is also implicated in renal injury and fibrosis when dysregulated (Li et al., 2020). The renal angiopoietin-like protein 4 (ANGPTL4), a key protein involved in lipoprotein metabolism, interacts with Integrin β1 and exert fibrogenic effect in diabetic kidneys (Srivastava et al., 2024).

On the protective side, The activation of the endothelial glucocorticoid receptor (GR) exerts anti-inflammatory effects. The db/db mice lacking endothelial GR showed more severe fibrosis in multiple organs (Srivastava and Goodwin, 2023). The fibrogenic phenotype in the kidneys of diabetic mice lacking endothelial GR is associated with aberrant cytokine and chemokine reprogramming, augmented Wnt signaling and suppression of fatty acid oxidation, which demonstrated that endothelial GR is an essential antifibrotic molecule in diabetes (Srivastava et al., 2021). FGFR1 signaling protects against kidney fibrosis by inhibiting EndMT and suppressing TGF-β/smad signaling (Li et al., 2017). SIRT3-mediated mechanisms in endothelial cells improve mitochondrial function.The suppression in SIRT3 was associated with the induction of TGF-β/smad signaling, higher level of HIF1a accumulation and PKM2 dimer formation, led to abnormal glycolysis and linked abnormal mesenchymal transformations (Srivastava et al., 2018).

The development of drugs for DKD is also in full swing, several drugs have been evaluated for their unique therapeutic mechanisms: Linagliptin, a DPP-4 inhibitor, enhances incretin hormone activity, reducing hyperglycemia and exerting anti-inflammatory and antifibrotic effects (Nady et al., 2024). Empagliflozin, an SGLT2 inhibitor, improves DKD by inhibiting glycolysis, reducing intracellular glucose uptake, and lowering oxidative stress (Huang et al., 2024a). ACE inhibition and N-acetyl-seryl-aspartyl-lysyl-proline (AcSDKP) suppressed defective metabolism-linked mesenchymal transformations and reduced collagen-I and fibronectin accumulation in the diabetic kidneys (Srivastava et al., 2020). Mineralocorticoid antagonists like finerenone exert anti-inflammatory and antifibrotic effects, reducing proteinuria and slowing DKD progression. mice. Its effect was associated with reduced oxidative stress, mitochondrial fragmentation, and apoptosis while restoring the mitophagy via PI3K/Akt/eNOS signaling (Yao et al., 2023).

The significance of N6-methyladenosine (m6A) modification in the pathogenesis of DKD has become increasingly prominent. The m6A modification is a prevalent form of RNA methylation that plays a critical role in regulating various biological processes, including RNA stability, splicing, translation, and degradation (Oerum et al., 2021). This modification is added by methyltransferases known as “writers,” removed by demethylases termed “erasers,” and recognized by “readers” which facilitate the functional outcomes of m6A-modified RNA (Jiang et al., 2021). The core components involved in m6A modification include methyltransferase complexes (e.g., METTL3 and METTL14), demethylases (e.g., FTO and ALKBH5), and reader proteins (e.g., YTHDF and HNRNPA2B1). Recent studies have shown that m6A modification was involved in several essential physiological processes, such as stem cell differentiation, immune response, and metabolism (Shi et al., 2019). The dysregulation of m6A modification can be linked to various diseases, including cancer (He et al., 2019), obesity (Liu et al., 2021), and cardiovascular diseases (Xu et al., 2022), emphasizing its role in cellular homeostasis.

In the context of diabetic kidney disease (DKD), emerging evidence suggests that m6A modification plays a significant role in the pathogenesis and progression of renal damage (Ye et al., 2023; Huang et al., 2024b; Sun et al., 2023). Increased expression of METTL3 has been observed in renal tissues of diabetic models, which correlates with enhanced m6A methylation of specific target mRNAs involved in inflammation and fibrosis (Wang et al., 2024). Similarly, overexpression of m6A erasers like FTO has been associated with increased renal inflammation and fibrosis. Knockdown of FTO markedly increased SAA2 mRNA m6A modification and decreased SAA2 mRNA expression, thus alleviating the podocyte injury and inflammation (Lang et al., 2024).

This study aims to investigate the specific expression patterns of the m6A geneset in the pathogenesis of diabetic kidney disease (DKD) by integrating bulk RNA sequencing, single-cell and spatial transcriptomics, utilizing publicly available databases. The research seeks to identify core targets and uncover potential therapeutic agents, thereby providing novel insights for the clinical prevention and treatment of DKD.

## 2 Method

### 2.1 Date merge of bulk RNA transcriptomics and differential analysis

All datasets were downloaded from the Gene Expression Omnibus (GEO) database (http://www.ncbi.nlm.nih.gov/geo/). We used expression profiling by high throughput sequencing (GSE142025,GSE162830,GSE199838,GSE154881,GSE175759) (Fan et al., 2019; Eadon et al., 2021; Park et al., 2022) for initial analysis, including 45 control samples and 56 DKD samples. Detailed platform and annotation information for these datasets were provided in Supplementary Table S1. The R package “sva” was used to merge these data cohorts and address batch effects, with the ComBat function used for batch effect adjustment. The differential analysis was conducted with log2FC > 1.5 and P-value <0.05 (Wei et al., 2022). The significantly enriched m6a gene would be designated as differentially expressed genes (DEGs). The detailed m6a regulation gene was shown in Supplementary Figure S1. The GO, KEGG, GSEA/GSVA analysis was used to explore the potential pathway.

### 2.2 Screening of hub gene based on machine leaning

All 28 m6a regulation genes were input into 3 machine learning algorithms: the Least Absolute Shrinkage and Selection Operator (LASSO), the Regularized Random Forest (RRF)and the Extreme Gradient Boosting (XGBoost) regression (Feature importance and SHAP explanation). The m6a genes with both favourable machine learning algorithm characteristics and DEG properties would be screened as hub genes.

### 2.3 Validation of hub gene

The chosen hub gene would undergo validation in an external dataset (GSE99339, GSE30529, GSE96804, GSE104954, and GSE30528, detailed in Supplementary Table S1). The receiver operating characteristic (ROC) analysis was executed. Area under the Curve (AUC) values exceeding 0.65 were deemed to exhibit commendable diagnostic efficacy (Shen et al., 2024). Correlations between the expression levels of hub gene and clinical indicators among patients diagnosed with DKD were investigated using Nephroseq (http://v5.nephroseq.org).

### 2.4 Single cell transcriptome analysis of LRPPRC

The expression level of LRPPRC in single cell transcriptome data would be conducted in two different platform. Firstly, the gene would be imported in the Kidney Integrative Transcriptomics (K.I.T.) platform (http://humphreyslab.com/SingleCell/) based on the original data from Human Diabetic Kidney (23980 nuclei) (Wilson et al., 2019) and Human DKD scATAC-seq (Wilson et al., 2022). Secondly, 6 signature DKD samples and 7 control samples from the GSE195460 and GSE131882 dataset would be aggregated and analyzed. The “Seurat” package was used for preprocessing and clustering. The cell pseudotime analysis was conducted using the BiocGenerics and monocle packages.

### 2.5 Spatial transcriptomics of LRPPRC

The spatial transcriptomics data originated from GSE261545 (Isnard et al., 2024). Detailed information was provided in Supplementary Table S1. The conditional autoregressive-based deconvolution (CARD) was applied for spatial division and inter-regional difference analysis. The LRPPRC would be mapped to specific locations in pathological sections to reflect its actual expression.

### 2.6 Filter of potential compounds

The PPI map of 28 m6a genes was constructed based on the STRING platform to determine the location of LRPPRC, and then possible compounds were screened based on the DSigDB database, mainly regulating LPRRC and supplemented by other m6a regulatory functions.

## 3 Result

### 3.1 Landscape of differential genes and enrichment pathway in DKD

**FIGURE 1:**
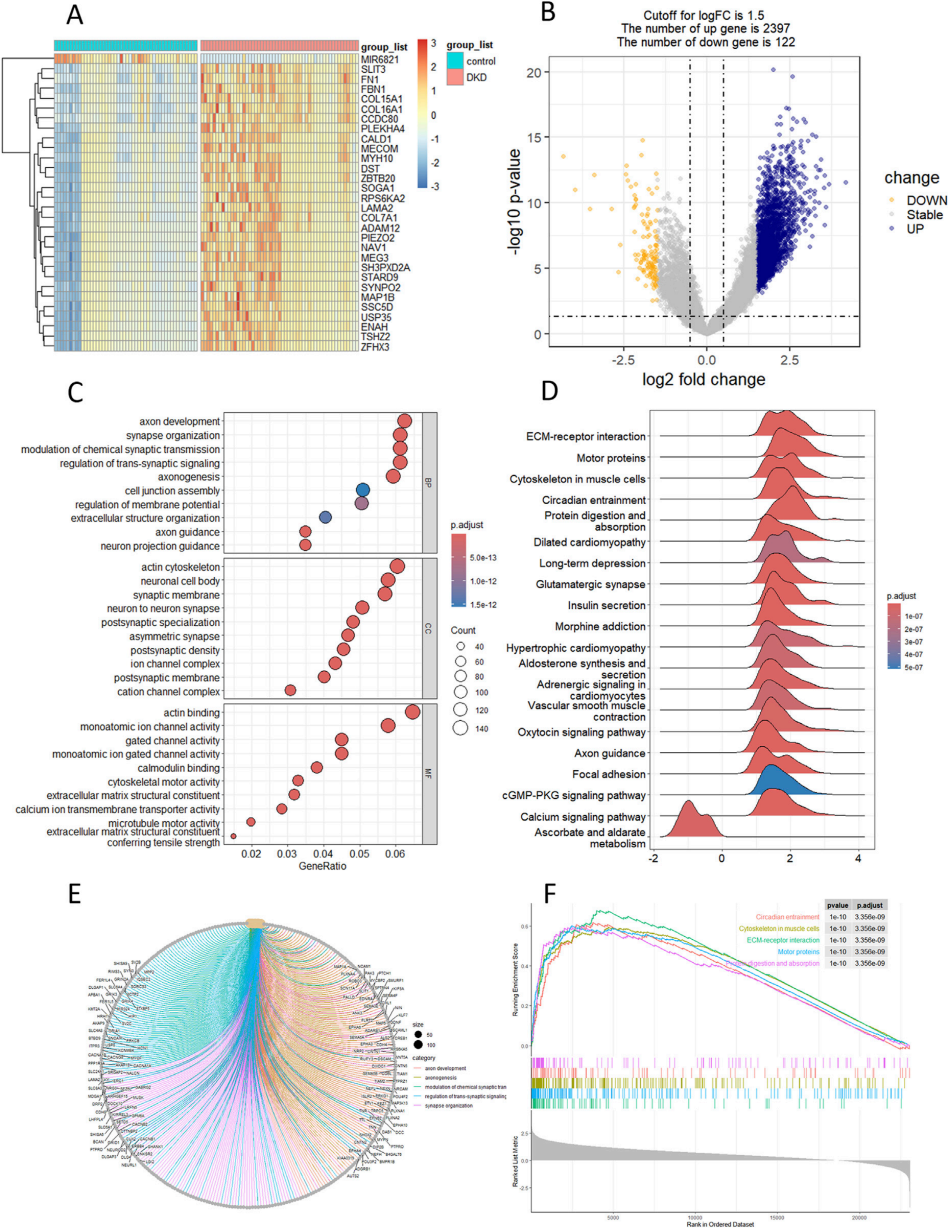
Landscape of differential genes and pathway in DKD. **(A, B)** Heatmap and volcano plot of DEGs between control and DKD groups. **(C)** GO enrichment analysis of DEGs. **(D)** GSEA analysis of hallmark genesets. **(E)** KEGG analysis. **(F)** Top-5 pathway based on GSEA analysis.

The results of the DKD bulk RNA transcriptome analysis were shown in Figure 1. Data preprocessing was shown in Supplementary Figure S2. The heatmap showed that most of the top 30 DEGs were upregulated genes (Figure 1A). The volcano plot indicated 2397 upregulated and 122 downregulated genes (Figure 1B). The pathway enriched were most related with renal fibrosis like “extracellular matrix structural constituent” and “regulation of membrane potential” (Figure 1C). GSEA has revealed the potential m6a-associated pathways such as cGMP-PKG pathway (Figure 1D). KEGG highlighted alterations in the signaling of ECM-receptor interaction that accompany DKD (especially the renal fibrosis) (Figure 1E). Among the top five activated pathways, 2 were regulated by m6a regulation (ECM-receptor interaction, Protein digestion and absorption) (Figure 1F).

### 3.2 The differential gene analysis and GSVA analysis based on m6a geneset in DKD

**FIGURE 2:**
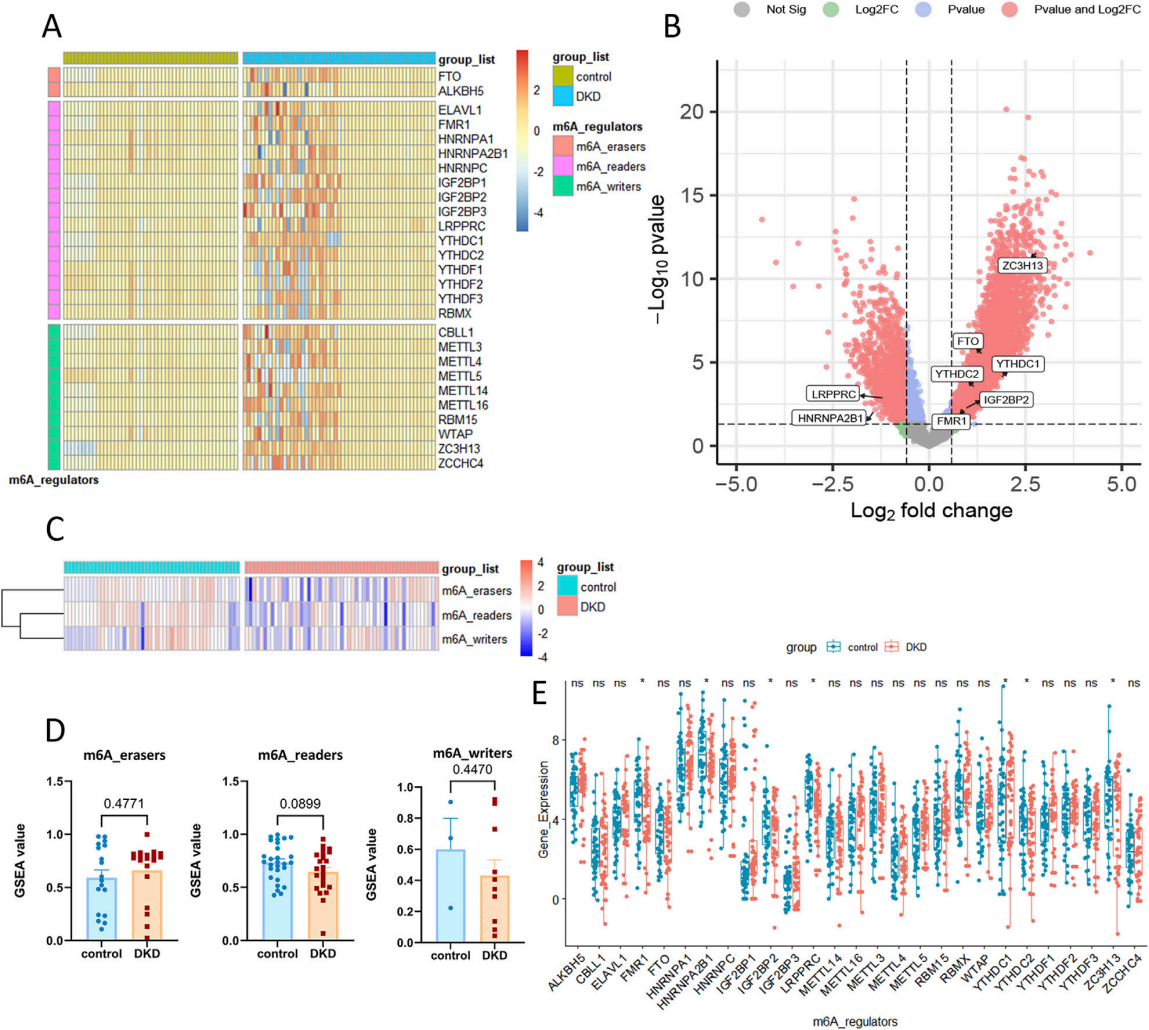
The differential gene analysis and GSVA analysis based on m6a geneset in DKD. **(A, B)** Heatmap and volcano plot of m6a geneset between control and DKD groups. **(C, D)** GSVA analysis of 3 m6a regulation. **(E)** The detailed expression of 28 m6a gene.

The heatmap and volcano plot (Figures 2A, B) showed the m6a genes which expressed deferentially in DKD patients. GSVA showed that the two types of m6a regulation, namely m6a-readers and m6a-writers, were generally suppressed in DKD patients (Figure 2C). Moreover, the inhibition trend of m6a-readers was more obvious (Figure 2D). The t-test of independent samples showed that FMR1, HNRNPA2B1, IGF2BP2, LRPPRC, YTHDC2, and ZC3H13 were significantly inhibited in m6a geneset (Figure 2E).

### 3.3 The output of machine learning

**FIGURE 3:**
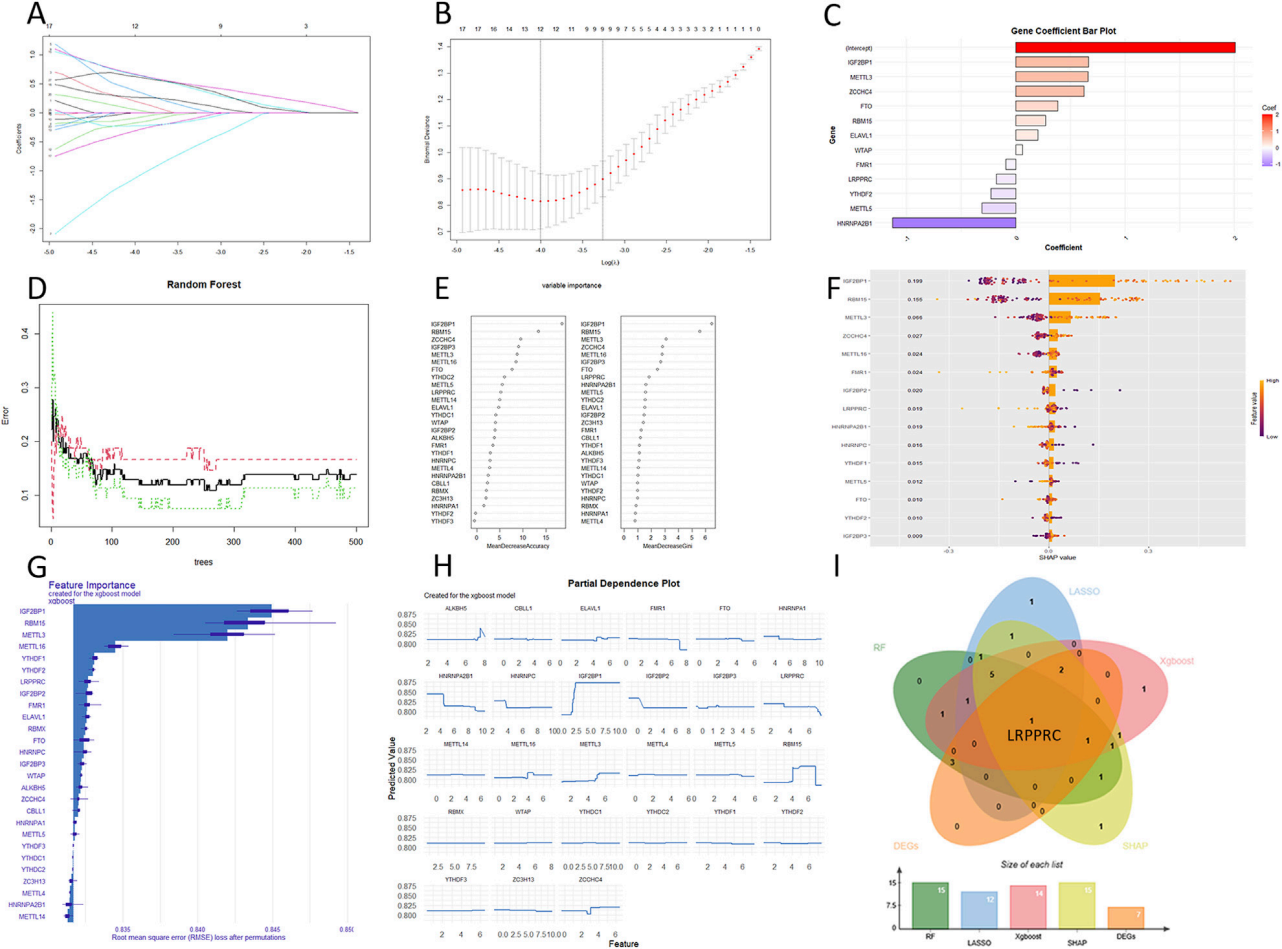
The output of 3 machine learning algorithms. **(A–C)** LASSO regression. **(D, E)** RRF. **(F)** SHAP for XGBoost regression. **(G, H)** Feature imprtace and partial dependence for XGBoost regression. **(I)** Intersetion of machine learning and DEGs.

In the LASSO regression, most m6a genes were positively correlated with DKD (Figure 3A). 12 genes were selected for output (Figure 3B) and ranked by correlation coefficient (Figure 3C). The RFF analysis used 500 decision trees (Figure 3D). 15 genes were selected for output and all ranked by accuracy value and Gini value (Figure 3E). SHAP for XGBoost regression showed the top variables as GF2BP1, RBM15 and METTL3 (Figure 3F). The feature importance and partial dependence of XGBoost showed the similar output (Figures 3G, H). The intersection of all results indicated that LRPPRC as the hub gene (Figure 3I). In validation dataset, LRPPRC was also significantly downregulated in the DKD patient. ROC analysis showed an AUC of 0.878 for LRPPRC (Supplementary Figure S3).

### 3.4 The expression and distribution of LRPPRC in single cell and spatial district of DKD

**FIGURE 4:**
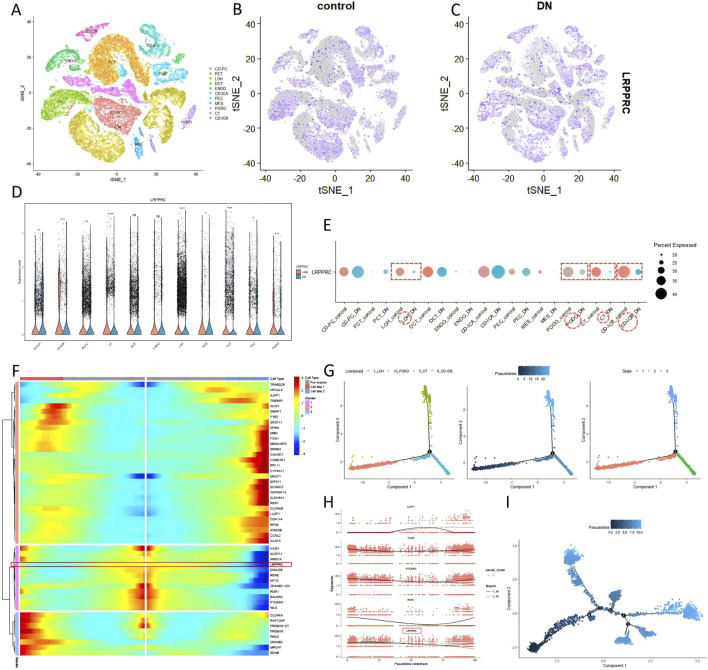
Expression of LRPPRC in DKD single-cell (GSE195460 and GSE131882). **(A–E)** The quantitative analysis of LRPPRC. **(F–I)** The pseudotime analysis.

The quality control results of the single-cell dataset were shown in Supplementary Figure S4. Low heterogeneity was observed between samples, with no interference from mitochondrial, ribosomal, or erythrocyte RNA. A total of 59,098 nuclei and 35,618 genes were included. In the DKD group, the combined data were effectively reduced to 13 clusters and 11 cell types: collecting duct-principal cell (CD-PC), proximal convoluted tubular cell (PCT), Loop of Henle cell (LOH), distal convoluted tubular cell (DCT), convoluted tubular cell (CT), collecting duct-intercalated cell type A (CD-ICA), collecting duct-intercalated cell type B(CD-ICB), glomerular parietal epithelial cell (PEC), endothelial cell (ENDO), mesenchymal cell (MES), podocyte (PODO) (Figure 4A). LRPPRC was broadly expressed across all renal cell types and was downregulated in patients with diabetic kidney disease (Figures 4B, C). Particularly in the LOH, PODO, CT, and CD-ICB cell populations, it exhibited significantly lower expression levels (Figures 4D, E). Upon extracting the LOH, PODO, CT, and CD-ICB cell subpopulations from DKD patients, pseudotime analysis revealed that these four subpopulations primarily were clustered into three time points (Figure 4G). Given that the apoptosis of LOH cells representing early renal injury, LOH subpopulation was therefore designated as the starting point for differentiation. The trajectory illustrated a common differentiation direction between LOH and CT towards PODO, suggesting that as DKD progresses, tubular injury progressively impacted the podocytes in the glomeruli. A dynamic analysis of the top fifty differentially expressed genes revealed that the majority of these genes were upregulated in accordance with the direction of cell differentiation and disease progression, while LRPPRC exhibited the opposite trend, decreasing over time (Figure 4F), particularly pronounced in LOH cells (Figure 4H). In the pseudotime analysis of the LOH cell across all samples, LOH cells exhibited five distinct differentiation nodes, with a trajectory that was more diverse in DKD patients (Figure 4I).

**FIGURE 5:**
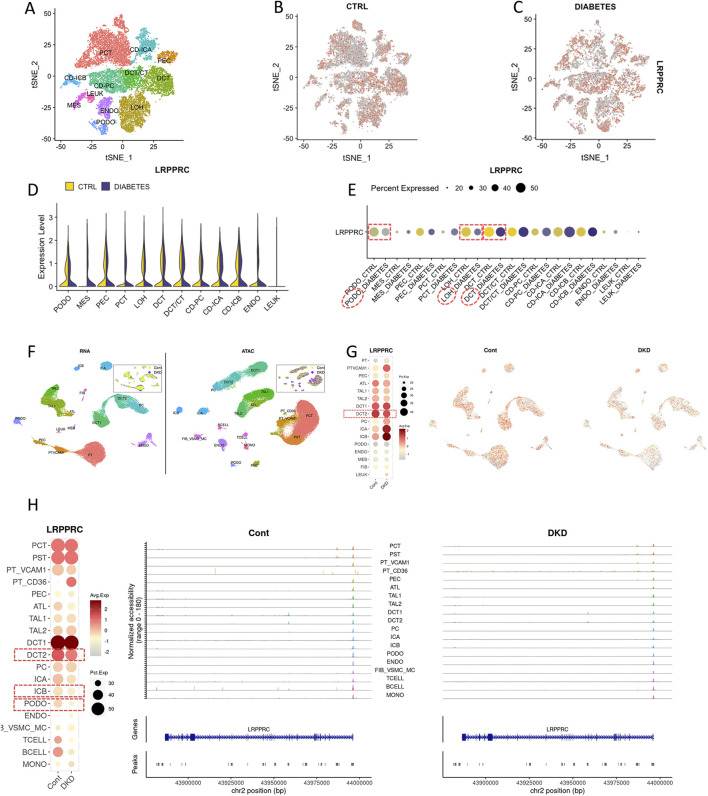
Expression of LRPPRC in DKD single-cell (K.I.T database). **(A–E)** LRPPRC in DKD single-cell. **(F–H)** LRPPRC in ATAC-seq.

In the K.I.T single-cell online platform, kidney cells were divided into 12 subgroups (Figure 5A). The LRPPRC was widely expressed (Figure 5B), and the expression of LRPPRC was downregulated in DKD patients compared with healthy people (Figure 5C). The main renal cells with downregulated LRPPRC expression were PODO,LOH, and DCT (Figures 5D, E). ATAC-seq analysis showed that renal cell chromatin was grouped into 20 subgroups (Figure 5F). Compared with single cells, DCT cells were subdivided into two subgroups, DCT1 and DCT2. The PT (Proximal Tubule cells) cells were subdivided into PT_CD36 and PT_VCAM1. LOH cells are subdivided into TAL (Thick Ascending Limb) and ATL (Ascending Thin Limb). The more immune cells were distinguished, such as B cells and T cells. Fib_VSMC_MC stand for a mixture of Fibroblasts, Vascular Smooth Muscle Cells and Myofibroblasts. Gene expression showed that the distribution of LRPPRC in the open region of the genome of DKD patients was lower than that of healthy people, and it was mainly downregulated in the DCT2 subgroup (Figure 5G). In terms of gene activity and accessibility, LRPPRC was mainly downregulated in DCT2, ICB and PODO cells. It was also generally downregulated in immune cells (B cell, T cell) (Figure 5H).

**FIGURE 6:**
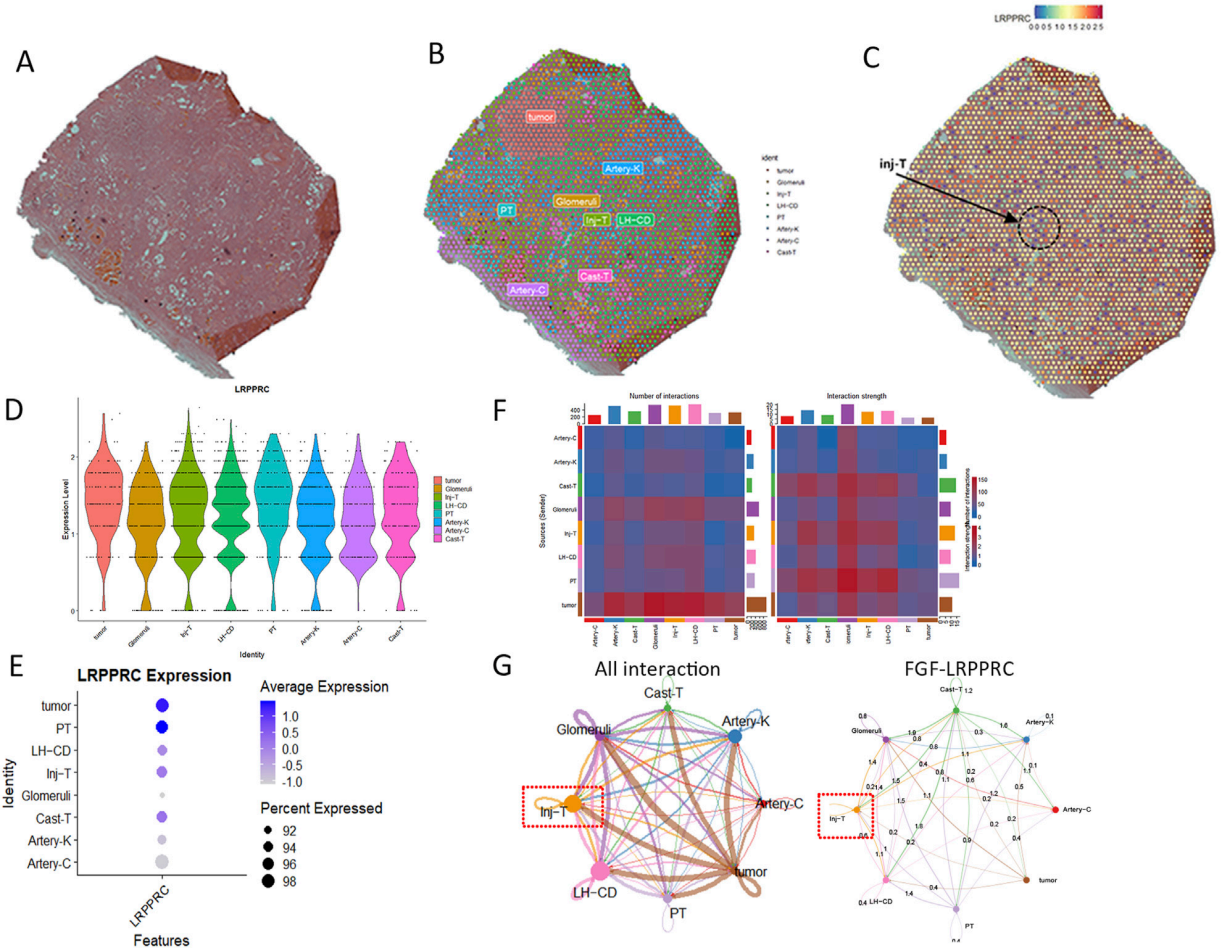
Expression of LRPPRC in spatial district of DKD (GSE261545). **(A)** raw kidney tissue slides. **(B)** The annotation of spatial section. **(C)** The distribution of LRPPRC in spatial section. **(D)** The violin plot of LRPPRC quantity in spatial section. **(E)** The dot plot of LRPPRC quantity in spatial section. **(F)** The overall cell chat between spatial section. **(G)** The role of FGF-LRPPRC in cell communication.

The high-resolution section of kidney tissue biopsy from DKD patients was shown in Figure 6A. The spatial transcriptome included a total of 18,085 genes and 2802 sampling points. The section could be divided into 5 regions: glomeruli, injured renal tubules (Inj−T), arteries within the capsule (Artery−C), Loop of Henle and collecting ducts (LH−CD), proximal tubules (PT), tubules with endoluminal protein casts (cast-T), arteries within the renal parenchyma (arterion-K) and tumor (Figure 6B). The low activity of LRPPRC was mainly detected in the injured renal tubules (Figure 6C). The low quantitative expression of LRPPRC was reflected on glomeruli and injured renal tubules (Figures 6D, E). In all renal regions, except tumor tissue, the glomeruli produced the most cell communication, while inj-T produced the strongest cell communication (Figure 6F). The inj-T strongly interacted with all other renal regions, among which FGF-LRPPRC pathway was the most widely distributed one (Figure 6G).

### 3.5 The expression of LRPPRC mRNA in clinical patients

**FIGURE 7:**
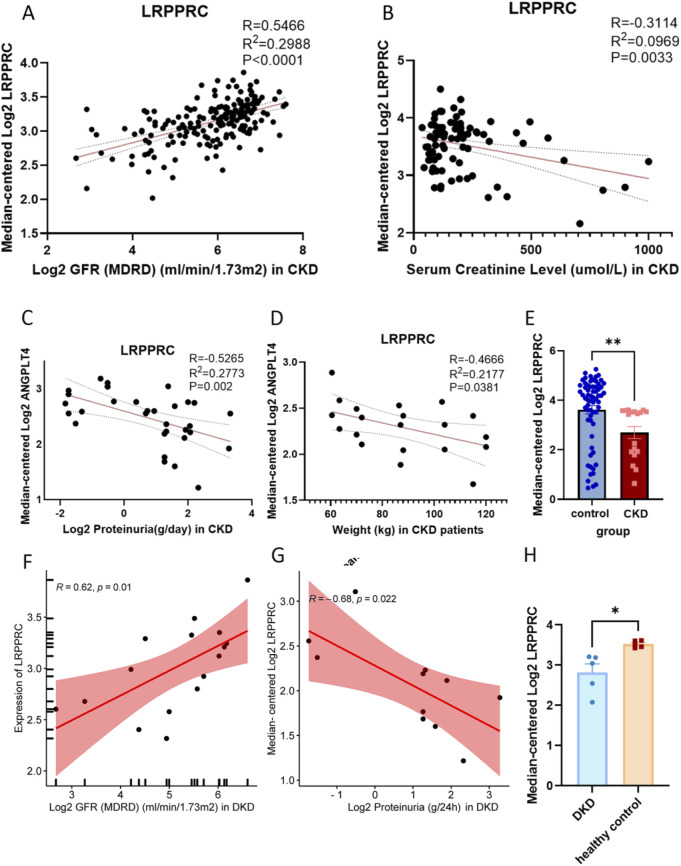
The expression of LRPPRC mRNA in clinical patients. **(A)** The association between LRPPRC mRNA and GFR in CKD patients. **(B)** The association between LRPPRC mRNA and serum creatinine level in CKD patients. **(C)** The association between LRPPRC mRNA and proteinuria in CKD patients. **(D)** The association between LRPPRC mRNA and weight in CKD patients. **(E)** The expression of LRPPRC mRNA in CKD patients. **(F)** The association between LRPPRC mRNA and GFR in DKD patients. **(G)** The association between LRPPRC mRNA and proteinuria in DKD patients. **(H)** The expression of LRPPRC mRNA in DKD patients. *P < 0.05, unpaired t-test method, **P < 0.01, unpaired t-test method.

Utilizing the hub gene LRPPRC as the retrieval keyword, relevant clinical data of Chronic Didney Disease (CKD) and Diabetic Kidney Disease (DKD) in the Nephroseq platform were scrutinized. Following correction and summarization, it was observed that the expression levels of LRPPRC mRNA in CKD patients were notably downregulated, exhibiting significant positive correlations with Glomerular Filtration Rate (GFR), the significant negative correlations with serum creatinine, proteinuria and weight (Figures 7A–E). Similarly, LRPPRC was downregulated in DKD patients, pisitively correlated with GFR and negatively correlated with proteinuria. (Figures 7F–H).This means that the worse the kidney function, the lower the expression of LRPPRC mRNA.

### 3.6 The filter of compounds targeting m6a regulation

**FIGURE 8:**
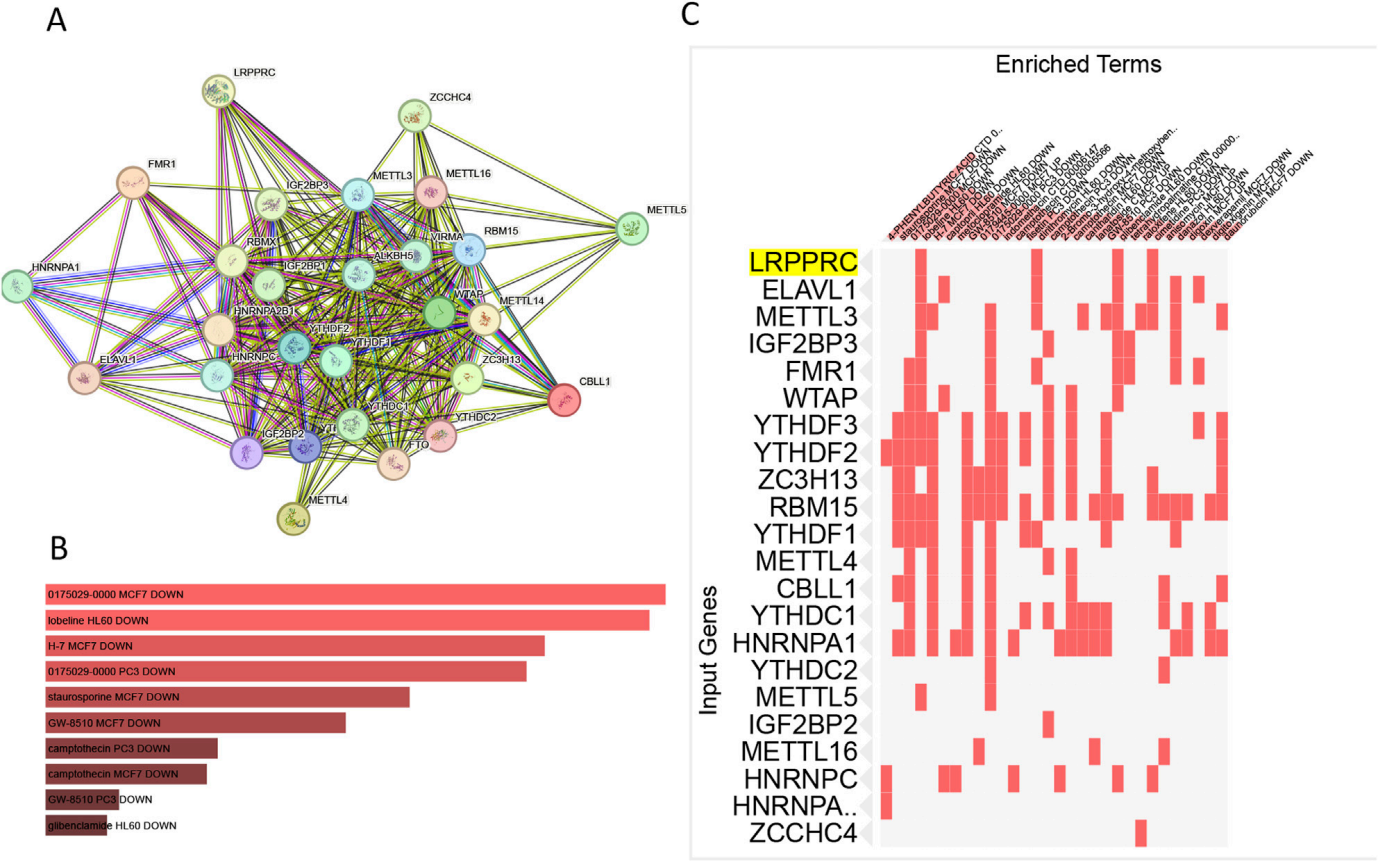
The filter of compounds targeting m6a regulation.**(A)** PPI map based on m6a geneset. **(B)** Top 10 drugs that interfere with the m6a gene. **(C)** Drugs targeting LRPPRC gene.

The PPI network with LRPPRC as the core was shown in Figure 8A. The 28 m6a genes were input into the drug screening platform with filtering conditions of P < 0.05 and Odds Ratio >5. The top 10 drug candidates were shown in Figure 8B, namely 0175029-0000(in both MCF7 and PC-3 cell line), lobeline, H-7, staurosporine, GW-8510, camptothecin (in both MCF7 and PC-3 cell line), GW-8510, glibenclamide, 4-PHENYLBUTYRIC ACID and fisetin. All drugs showed a tendency to inhibit the expression of m6a gene, which can be specifically seen in Supplementary Table S2. There were four compounds targeting LRPPRC as shown in Figure 8C lobeline, dirithromycin, glibenclamide and cimetidine. lobeline was considered as an important potential compound involved in the regulation of LRPPRC and other m6a genes (YTHDF1; YTHDF2; RBM15; YTHDF3; WTAP; FMR1; METTL3; METTL5; IGF2BP3; ELAVL1).

## 4 Discussion

Our study evaluated the expression patterns of the m6a geneset in the pathogenesis of DKD. GSEA has revealed the potential m6a-associated pathways such as cGMP-PKG pathway. GSVA showed that the two types of m6a regulation, namely m6a-readers and m6a-writers, were generally suppressed in DKD patients.

The m6A writers include a group of methyltransferases, with the methyltransferase-like 3 (METTL3) and methyltransferase-like 14 (METTL14) being the most well-characterized. They work together to catalyze the addition of methyl groups to the adenosine residues in RNA (Ries et al., 2019). Although some m6A writers, such as METTL5 and ZC3H proteins, are not directly involved in the regulation of m6a methylation (Turkalj and Vissers, 2022), However, some studies have shown that they recruit the m6A methyltransferase complex to target mRNA, so they were widely included in bioinformatics research (Huang et al., 2022; Jiang et al., 2023). The m6A readers include proteins such as YTH domain-containing proteins (YTHDF1, YTHDF2, and YTHDF3), HNRNPA2B1, and others that recognize and bind to m6A-modified RNA. These readers play pivotal roles in interpreting the m6A signals, regulating RNA fate, and thereby influencing gene expression (Lee et al., 2021).In DKD, the suppression of m6A writers and readers suggests a dysregulation in RNA metabolism. This disorder will lead to epithelial-to-mesenchymal transition (EMT) and aggravate the progression of renal fibrosis by regulating TGF-β signaling pathway, affecting the stability of lncRNA and miRNA, and activating the expression of extracellular matrix-related genes (Liu, 2024).

The output of 3 machine learning algorithm and differential analysis has determined the LRPPRC as the hub gene. LRPPRC (Leucine-Rich Pentatricopeptide Repeat Containing Protein) is a multifunctional protein that plays crucial roles in RNA metabolism, mitochondrial function, and cellular stress responses. It is primarily involved in the stabilization, transport, and translation of mitochondrial mRNAs, ensuring proper mitochondrial protein synthesis, which is vital for energy metabolism and overall cellular health (Liu and McKeehan, 2002).The knockout of LRPPRC in mice causes an additive effect on mitochondrial translation leading to embryonic lethality and reduced growth of mouse embryonic fibroblasts (Rubalcava-Gracia et al., 2024). In T2DM, the expression of LRPPRC was differentially expressed in pancreatic, liver, adipose tissue, blood and skeletal muscle (Li et al., 2024). The predictive model containing LRPPRC (AUC = 0.806) were used as the diagnostic basis of diabetic retinopathy (Wang et al., 2023). In the context of DKD, studies have indicated that the downregulation of LRPPRC may disrupt mitochondrial mRNA stability and translation, leading to mitochondrial dysfunction (Ruzzenente et al., 2012). Mitochondrial impairment is associated with increased oxidative stress, which can exacerbate kidney injury through various mechanisms, including inflammation, fibrosis, and apoptosis of renal cells (Liu et al., 2024). In our study, expression levels of LRPPRC mRNA were notably downregulated in DKD patients, exhibiting significant positive correlations with GFR, suggesting its protective effect on kidney function.

LRPPRC was downregulated in the LOH, PODO, CT, and CD-ICB cell populations, most of which were tubular cells. It exhibited the decreasing trend over time, particularly pronounced in LOH cells. The Loop of Henle (LOH) is a critical segment of the kidney and plays a crucial role in urine concentration and electrolyte balance, relying heavily on efficient mitochondrial function (Palm and Carlsson, 2005). The downregulation of LRPPRC in LOH cells may disrupt mitochondrial mRNA stability and exacerbate renal injury. The pseudotime analysis revealed that LRPPRC was a crucial gene during the transition from tubular injury to podocyte damage and a key gene involved in the interaction between injured tubular cells and other types of renal cells. This finding may suggested an early warning role of LRPPRC in the progression of DKD. Traditionally, DKD has been regarded as a disease primarily characterized by glomerular lesions (Wang and Chen, 2022). However, an increasing body of evidence in recent years has demonstrated that changes in tubular function may represent the “driving force” behind the development of DKD (Chatterjee et al., 2025). Tubular injury not only drives the progression of DKD but also exacerbates pre-existing glomerular damage. Some scholars have proposed the “tubule-centric hypothesis” (Zeni et al., 2017), which posits that tubular injury and functional alterations may better explain the pathogenesis and progression of DN. As a potential biomarker of injured renal tubules, LRPPRC is significantly associated with urinary protein levels and glomerular filtration rate in clinical DKD patients, potentially providing a predictive window for early tubular lesions in DKD.

Finally, the lobeline might be an important potential compound involved in the regulation of LRPPRC and other m6a genes. As a compound derived from the plant Lobelia inflata, lobeline has been shown to have various biological effects, including modulation of neurotransmitter release and potential therapeutic benefits in respiratory and neurodegenerative disorders (Remya et al., 2023). At present, there was a lack of research on this drug for endocrine or kidney disease, and its actual efficacy needs to be verified *in vivo* or *in vitro*.

## 5 Conclusion

In summary, we analyzed the landscape of m6A modification regulators in DKD development and progression. The m6a-readers and m6a-writers, were generally suppressed in DKD patients. the LRPPRC was identified as the hub gene and revealed the good diagnostic potential for eGFR in DKD. The lobeline might be an important potential compound involved in the regulation of LRPPRC and other m6a genes.

## Data Availability

The datasets presented in this study can be found in online repositories. The names of the repository/repositories and accession number(s) can be found in the article/supplementary material.

## References

[B1] Akhtar M. Taha N. M. Nauman A. Mujeeb I. B. Al-Nabet A. D. M. H. (2020). Diabetic kidney disease: past and present. Adv. Anat. Pathol. 27 (2), 87–97. 10.1097/PAP.0000000000000257 31876542

[B2] Barutta F. Bellini S. Canepa S. (2022). Novel biomarkers of diabetic kidney disease: current status and potential clinical application. Acta Diabetol. 59 (3), 439–441. 10.1007/s00592-021-01816-5 35092493

[B3] Chatterjee A. Tumarin J. Prabhakar S. (2025). Cellular cross-talk drives mesenchymal transdifferentiation in diabetic kidney disease. Front. Med. (Lausanne) 11, 1499473. 10.3389/fmed.2024.1499473 39839616 PMC11747801

[B4] Eadon M. T. Lampe S. Baig M. M. Collins K. S. Melo Ferreira R. Mang H. (2021). Clinical, histopathologic and molecular features of idiopathic and diabetic nodular mesangial sclerosis in humans. Nephrol. Dial. Transpl. 37 (1), 72–84. 10.1093/ndt/gfaa331 PMC8826640 33537765

[B5] Fan Y. Yi Z. D'Agati V. D. Sun Z. Zhong F. Zhang W. (2019). Comparison of kidney transcriptomic profiles of early and advanced diabetic nephropathy reveals potential new mechanisms for disease progression. Diabetes 68 (12), 2301–2314. 10.2337/db19-0204 31578193 PMC6868471

[B6] Hadpech S. Thongboonkerd V. (2024). Epithelial-mesenchymal plasticity in kidney fibrosis. Genesis 62 (1), e23529. 10.1002/dvg.23529 37345818

[B7] He L. Li H. Wu A. Peng Y. Shu G. Yin G. (2019). Functions of N6-methyladenosine and its role in cancer. Mol. Cancer 18 (1), 176. 10.1186/s12943-019-1109-9 31801551 PMC6892141

[B8] Huang J. Liu Y. Shi M. Zhang X. Zhong Y. Guo S. (2024a). Empagliflozin attenuating renal interstitial fibrosis in diabetic kidney disease by inhibiting lymphangiogenesis and lymphatic endothelial-to-mesenchymal transition via the VEGF-C/VEGFR3 pathway. Biomed. Pharmacother. 180, 117589. 10.1016/j.biopha.2024.117589 39418962

[B9] Huang J. Yang F. Liu Y. Wang Y. (2024b). N6-methyladenosine RNA methylation in diabetic kidney disease. Biomed. Pharmacother. 171, 116185. 10.1016/j.biopha.2024.116185 38237350

[B10] Huang Q. Mo J. Liao Z. Chen X. Zhang B. (2022). The RNA m6A writer WTAP in diseases: structure, roles, and mechanisms. Cell Death Dis. 13 (10), 852. 10.1038/s41419-022-05268-9 36207306 PMC9546849

[B11] Isnard P. Li D. Xuanyuan Q. Wu H. Humphreys B. D. (2024). Histopathologic analysis of human kidney spatial transcriptomics data: toward precision pathology. Am. J. Pathol. 195, 69–88. 10.1016/j.ajpath.2024.06.011 39097165 PMC11686452

[B12] Jiang T. Xing L. Zhao L. Ye Z. Yu D. Lin S. (2023). Comprehensive analysis of m6A related gene mutation characteristics and prognosis in colorectal cancer. BMC Med. Genomics 16 (1), 105. 10.1186/s12920-023-01509-8 37194014 PMC10186803

[B13] Jiang X. Liu B. Nie Z. Duan L. Xiong Q. Jin Z. (2021). The role of m6A modification in the biological functions and diseases. Signal Transduct. Target Ther. 6 (1), 74. 10.1038/s41392-020-00450-x 33611339 PMC7897327

[B14] Lang Y. Wang Q. Sheng Q. Lu S. Yang M. Kong Z. (2024). FTO-mediated m6A modification of serum amyloid A2 mRNA promotes podocyte injury and inflammation by activating the NF-κB signaling pathway. FASEB J. 38 (2), e23409. 10.1096/fj.202301419RR 38193628

[B15] Lee J. H. Wang R. Xiong F. Krakowiak J. Liao Z. Nguyen P. T. (2021). Enhancer RNA m6A methylation facilitates transcriptional condensate formation and gene activation. Mol. Cell 81 (16), 3368–3385.e9. 10.1016/j.molcel.2021.07.024 34375583 PMC8383322

[B16] Li B. Zhu C. Dong L. Qin J. Xiang W. Davidson A. J. (2020). ADAM10 mediates ectopic proximal tubule development and renal fibrosis through Notch signalling. J. Pathol. 252 (3), 274–289. 10.1002/path.5517 32715474 PMC7702158

[B17] Li J. Shi S. Srivastava S. P. Kitada M. Nagai T. Nitta K. (2017). FGFR1 is critical for the anti-endothelial mesenchymal transition effect of N-acetyl-seryl-aspartyl-lysyl-proline via induction of the MAP4K4 pathway. Cell Death Dis. 8 (8), e2965. 10.1038/cddis.2017.353 28771231 PMC5596544

[B18] Li Y. L. Zhang Y. Chen N. Yan Y. X. (2024). The role of m6A modification in type 2 diabetes: a systematic review and integrative analysis. Gene 898, 148130. 10.1016/j.gene.2024.148130 38181926

[B19] Liu D. F. Chen X. J. He W. T. Lu M. Li Q. Zhang S. (2024). Update on the pathogenesis, diagnosis, and treatment of diabetic tubulopathy. Integr. Med. Nephrol. Androl. 11 (4), e23–e00029. 10.1097/IMNA-D-23-00029

[B20] Liu L. McKeehan W. L. (2002). Sequence analysis of LRPPRC and its SEC1 domain interaction partners suggests roles in cytoskeletal organization, vesicular trafficking, nucleocytosolic shuttling, and chromosome activity. Genomics 79 (1), 124–136. 10.1006/geno.2001.6679 11827465 PMC3241999

[B21] Liu S. Xiu J. Zhu C. Meng K. Li C. Han R. (2021). Fat mass and obesity-associated protein regulates RNA methylation associated with depression-like behavior in mice. Nat. Commun. 12 (1), 6937. 10.1038/s41467-021-27044-7 34836959 PMC8626436

[B22] Liu Y. H. (2024). Kidney fibrosis: fundamental questions, challenges, and perspectives. Integr. Med. Nephrol. Androl. 11 (4), e24–e00027. 10.1097/IMNA-D-24-00027

[B23] Nady M. E. Abd El-Raouf O. M. El-Sayed E. M. (2024). Linagliptin mitigates TGF-β1 mediated epithelial-mesenchymal transition in tacrolimus-induced renal interstitial fibrosis via smad/ERK/P38 and HIF-1α/LOXL2 signaling pathways. Biol. Pharm. Bull. 47 (5), 1008–1020. 10.1248/bpb.b23-00737 38797693

[B24] Oerum S. Meynier V. Catala M. Tisné C. (2021). A comprehensive review of m6A/m6Am RNA methyltransferase structures. Nucleic Acids Res. 49 (13), 7239–7255. 10.1093/nar/gkab378 34023900 PMC8287941

[B25] Palm F. Carlsson P. O. (2005). Thick ascending tubular cells in the loop of Henle: regulation of electrolyte homeostasis. Int. J. Biochem. Cell Biol. 37 (8), 1554–1559. 10.1016/j.biocel.2005.02.007 15896664

[B26] Park S. Lee H. Lee J. Cho S. Huh H. (2022). RNA-seq profiling of tubulointerstitial tissue reveals a potential therapeutic role of dual anti-phosphatase 1 in glomerulonephritis. J. Cell Mol. Med. 26 (12), 3364–3377. 10.1111/jcmm.17340 35488446 PMC9189340

[B27] Remya C. Dileep K. V. Variyar E. J. Omkumar R. V. Sadasivan C. (2023). Lobeline: a multifunctional alkaloid modulates cholinergic and glutamatergic activities. IUBMB Life 75 (10), 844–855. 10.1002/iub.2762 37335270

[B28] Ries R. J. Zaccara S. Klein P. Olarerin-George A. Namkoong S. Pickering B. F. (2019). m6A enhances the phase separation potential of mRNA. Nature 571 (7765), 424–428. 10.1038/s41586-019-1374-1 31292544 PMC6662915

[B29] Rubalcava-Gracia D. Bubb K. Levander F. Burr S. P. August A. V. Chinnery P. F. (2024). LRPPRC and SLIRP synergize to maintain sufficient and orderly mammalian mitochondrial translation. Nucleic Acids Res. 52, 11266–11282. 10.1093/nar/gkae662 39087558 PMC11472161

[B30] Ruzzenente B. Metodiev M. D. Wredenberg A. Bratic A. Park C. B. Cámara Y. (2012). LRPPRC is necessary for polyadenylation and coordination of translation of mitochondrial mRNAs. EMBO J. 31 (2), 443–456. 10.1038/emboj.2011.392 22045337 PMC3261557

[B31] Shen L. Xu X. Yue S. Yin S. (2024). A predictive model for depression in Chinese middle-aged and elderly people with physical disabilities. BMC Psychiatry 24 (1), 305. 10.1186/s12888-024-05766-4 38654170 PMC11040896

[B32] Shi H. Wei J. He C. (2019). Where, when, and how: context-dependent functions of RNA methylation writers, readers, and erasers. Mol. Cell. 74 (4), 640–650. 10.1016/j.molcel.2019.04.025 31100245 PMC6527355

[B33] Srivastava S. P. Goodwin J. E. (2023). Loss of endothelial glucocorticoid receptor accelerates organ fibrosis in db/db mice. Am. J. Physiol. Ren. Physiol. 325 (4), F519–F526. 10.1152/ajprenal.00105.2023 PMC10639025 37589053

[B34] Srivastava S. P. Goodwin J. E. Kanasaki K. Koya D. (2020). Metabolic reprogramming by N-acetyl-seryl-aspartyl-lysyl-proline protects against diabetic kidney disease. Br. J. Pharmacol. 177 (16), 3691–3711. 10.1111/bph.15087 32352559 PMC7393199

[B35] Srivastava S. P. Li J. Kitada M. Fujita H. Yamada Y. Goodwin J. E. (2018). SIRT3 deficiency leads to induction of abnormal glycolysis in diabetic kidney with fibrosis. Cell Death Dis. 9 (10), 997. 10.1038/s41419-018-1057-0 30250024 PMC6155322

[B36] Srivastava S. P. Zhou H. Setia O. Liu B. Kanasaki K. Koya D. (2021). Loss of endothelial glucocorticoid receptor accelerates diabetic nephropathy. Nat. Commun. 12 (1), 2368. 10.1038/s41467-021-22617-y 33888696 PMC8062600

[B37] Srivastava S. P. Zhou H. Shenoi R. Morris M. Lainez-Mas B. Goedeke L. (2024). Renal Angptl4 is a key fibrogenic molecule in progressive diabetic kidney disease. Sci. Adv. 10 (49), eadn6068. 10.1126/sciadv.adn6068 39630889 PMC11616692

[B38] Sun Y. Liu G. Li M. Wang L. He Z. Gu S. (2023). Study on the correlation between regulatory proteins of N6-methyladenosine and oxidative damage in cadmium-induced renal injury. Biol. Trace Elem. Res. 201 (5), 2294–2302. 10.1007/s12011-022-03345-w 35794303

[B39] Turkalj E. M. Vissers C. (2022). The emerging importance of METTL5-mediated ribosomal RNA methylation. Exp. Mol. Med. 54 (10), 1617–1625. 10.1038/s12276-022-00869-y 36266443 PMC9636144

[B40] Wang F. Bai J. Zhang X. Wang D. Xue J. (2024). METTL3/YTHDF2 m6A axis mediates the progression of diabetic nephropathy through epigenetically suppressing PINK1 and mitophagy. J. Diabetes Investig. 15 (3), 288–299. 10.1111/jdi.14113 PMC10906015 38013600

[B41] Wang X. Li X. Zong Y. Yu J. Chen Y. Zhao M. (2023). Identification and validation of genes related to RNA methylation modification in diabetic retinopathy. Curr. Eye Res. 48 (11), 1034–1049. 10.1080/02713683.2023.2238144 37529844

[B42] Wang Y. F. Chen H. Y. (2022). Ferroptosis in diabetic nephropathy: a narrative review. Integr. Med. Nephrol. Androl. 9 (1), 1. 10.4103/imna.imna_2_22

[B43] Wei Y. Wei H. Wei Y. Tan A. Chen X. Liao X. (2022). Screening and identification of human endogenous retrovirus-K mRNAs for breast cancer through integrative analysis of multiple datasets. Front. Oncol. 12, 820883. 10.3389/fonc.2022.820883 35265522 PMC8900282

[B44] Wilson P. C. Muto Y. Wu H. Karihaloo A. Waikar S. S. Humphreys B. D. (2022). Multimodal single cell sequencing implicates chromatin accessibility and genetic background in diabetic kidney disease progression. Nat. Commun. 13 (1), 5253. 10.1038/s41467-022-32972-z 36068241 PMC9448792

[B45] Wilson P. C. Wu H. Kirita Y. (2019). The single-cell transcriptomic landscape of early human diabetic nephropathy. Proc. Natl. Acad. Sci. U. S. A. 116(39):19619–19625. 10.1073/pnas 31506348 PMC6765272

[B46] Xu Z. Lv B. Qin Y. Zhang B. (2022). Emerging roles and mechanism of m6A methylation in cardiometabolic diseases. Cells 11 (7), 1101. 10.3390/cells11071101 35406663 PMC8997388

[B47] Yao L. Liang X. Liu Y. Li B. Hong M. Wang X. (2023). Non-steroidal mineralocorticoid receptor antagonist finerenone ameliorates mitochondrial dysfunction via PI3K/Akt/eNOS signaling pathway in diabetic tubulopathy. Redox Biol. 68, 102946. 10.1016/j.redox.2023.102946 37924663 PMC10661120

[B48] Ye W. Lv X. Gao S. Li Y. Luan J. Wang S. (2023). Emerging role of m6A modification in fibrotic diseases and its potential therapeutic effect. Biochem. Pharmacol. 218, 115873. 10.1016/j.bcp.2023.115873 37884198

[B49] Zeni L. Norden A. G. W. Cancarini G. Unwin R. J. (2017). A more tubulocentric view of diabetic kidney disease. J. Nephrol. 30 (6), 701–717. 10.1007/s40620-017-0423-9 28840540 PMC5698396

