# Supplementary material for: Landscape analysis of m6A modification regulators reveals LRPPRC as a key modulator in tubule cells for DKD: a multi-omics study
Source: Front Pharmacol. 2025 Apr 4;16:1506896. doi: 10.3389/fphar.2025.1506896 (PMC12006725; doi:10.3389/fphar.2025.1506896)
Supplement: Supplementary file 1 [file DataSheet1.docx]

Supplementary table 1 Characteristic of dataset included in the analysis

|  | Dataset | Data type | Platform | Sample | Tissue |
| --- | --- | --- | --- | --- | --- |
| Test dataset | GSE142025 | RNAseq | GPL17586 | 27 cases of DKD,9 cases of control | Kidney tissue |
|  | GSE162830 | RNAseq | GPL20301 | 18 cases of DKD,9 cases of control | Kidney tissue |
|  | GSE199838 | RNAseq | GPL21290 | 3 cases of DKD,1 cases of control | Kidney tissue |
|  | GSE154881 | RNAseq | GPL21290 | 5 cases of DKD,4 cases of control | Kidney tissue |
|  | GSE175759 | RNAseq | GPL16791 | 3 cases of DKD,22 cases of control | Kidney tissue |
|  | GSE131882 | snRNAseq | GPL24676 | 3 cases of DKD,3 cases of control | Kidney tissue |
|  | GSE195460 | snRNAseq | GPL24676 | 3 cases of DKD,4 cases of control | Kidney tissue |
|  | GSE261545 | Spatial Transcriptomic | GPL24676 | 1 case of DKD | kidney tissue |
| Validation dataset | GSE96804 | Expression profiling by array | GPL17586 | 41 cases of DKD,20 cases of control | Glomerulus |
|  | GSE30528 | Expression profiling by array | GPL571 | 9 cases of DKD,13 cases of control | Glomerulus |
|  | GSE30529 | Expression profiling by array | GPL571 | 10 cases of DKD,12 cases of control | Tubules |
|  | GSE104954 | Expression profiling by array | GPL24120  GPL22945 | 17 cases of DKD,21 cases of control | Tubules |
|  | GSE99339 | Expression profiling by array | GPL19184  GPL19109 | 14 cases of DKD,11 cases of control | Kidney tissue |

Supplementary table 2 The filter of compounds targeting m6a regulation

| Term | Overlap | P-value | Adjusted P-value | Odds Ratio | Combined Score | Genes |
| --- | --- | --- | --- | --- | --- | --- |
| 0175029-0000 MCF7 DOWN | 11/1488 | 2.45E-06 | 4.72E-04 | 8.102473217 | 104.6905265 | YTHDF1;YTHDF2;RBM15;YTHDF3;WTAP;YTHDC1;FMR1;METTL4;HNRNPA1;ZC3H13;CBLL1 |
| lobeline HL60 DOWN | 11/1510 | 2.82E-06 | 4.72E-04 | 7.974061139 | 101.8850956 | YTHDF1;YTHDF2;RBM15;YTHDF3;WTAP;FMR1;METTL3;METTL5;IGF2BP3;ELAVL1;LRPPRC |
| H-7 MCF7 DOWN | 10/1332 | 7.19E-06 | 7.05E-04 | 7.837451673 | 92.82167739 | YTHDF1;YTHDF2;RBM15;YTHDF3;YTHDC1;METTL3;METTL4;HNRNPA1;ZC3H13;CBLL1 |
| 0175029-0000 PC3 DOWN | 15/3326 | 8.45E-06 | 7.05E-04 | 5.806170574 | 67.82464846 | YTHDF1;YTHDF2;RBM15;YTHDF3;WTAP;YTHDC1;FMR1;YTHDC2;METTL3;METTL4;METTL5;IGF2BP3;HNRNPA1;ZC3H13;CBLL1 |
| staurosporine MCF7 DOWN | 7/649 | 2.40E-05 | 0.001600254 | 10.03634476 | 106.7796444 | YTHDF1;YTHDF2;RBM15;YTHDF3;HNRNPA1;ZC3H13;CBLL1 |
| GW-8510 MCF7 DOWN | 9/1290 | 4.23E-05 | 0.002355778 | 6.911500062 | 69.6006417 | YTHDF1;YTHDF2;RBM15;YTHDF3;YTHDC1;METTL4;HNRNPA1;ZC3H13;CBLL1 |
| camptothecin PC3 DOWN | 9/1494 | 1.33E-04 | 0.006096289 | 5.896969697 | 52.6519883 | YTHDF2;RBM15;YTHDF3;WTAP;YTHDC1;METTL4;IGF2BP3;IGF2BP2;ZC3H13 |
| camptothecin MCF7 DOWN | 9/1513 | 1.46E-04 | 0.006096289 | 5.816489362 | 51.36992239 | YTHDF2;RBM15;YTHDF3;WTAP;YTHDC1;METTL4;HNRNPA1;ZC3H13;CBLL1 |
| GW-8510 PC3 DOWN | 8/1312 | 3.19E-04 | 0.011848799 | 5.726380368 | 46.09418486 | YTHDF1;YTHDF2;RBM15;YTHDF3;YTHDC1;METTL3;HNRNPA1;ZC3H13 |
| glibenclamide HL60 DOWN | 8/1333 | 3.56E-04 | 0.01187858 | 5.629283019 | 44.70537009 | RBM15;WTAP;FMR1;METTL3;IGF2BP3;HNRNPC;ELAVL1;LRPPRC |
| 4-PHENYLBUTYRIC ACID CTD 00002847 | 3/108 | 4.55E-04 | 0.013805369 | 22.70514286 | 174.7374949 | YTHDF2;HNRNPA2B1;HNRNPC |
| fisetin PC3 DOWN | 5/541 | 8.34E-04 | 0.023224225 | 7.882868267 | 55.88002692 | YTHDF1;YTHDF2;RBM15;YTHDF3;YTHDC1 |


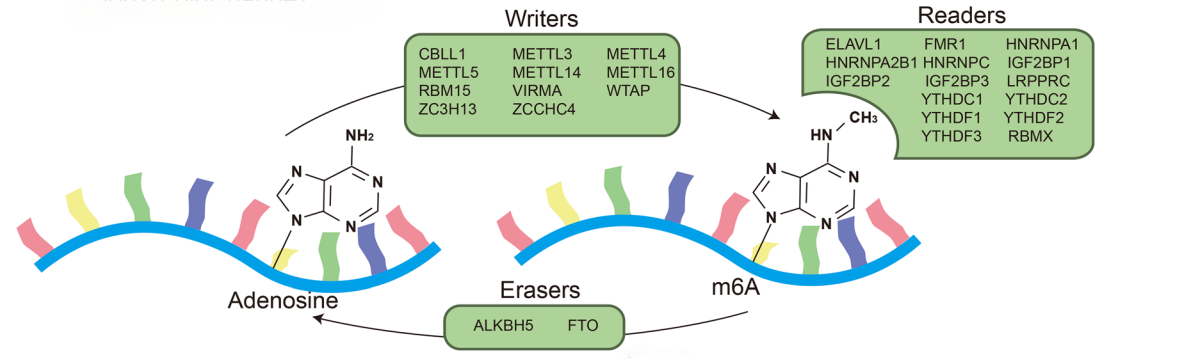


Supplementary figure 1. m6A regulation gene


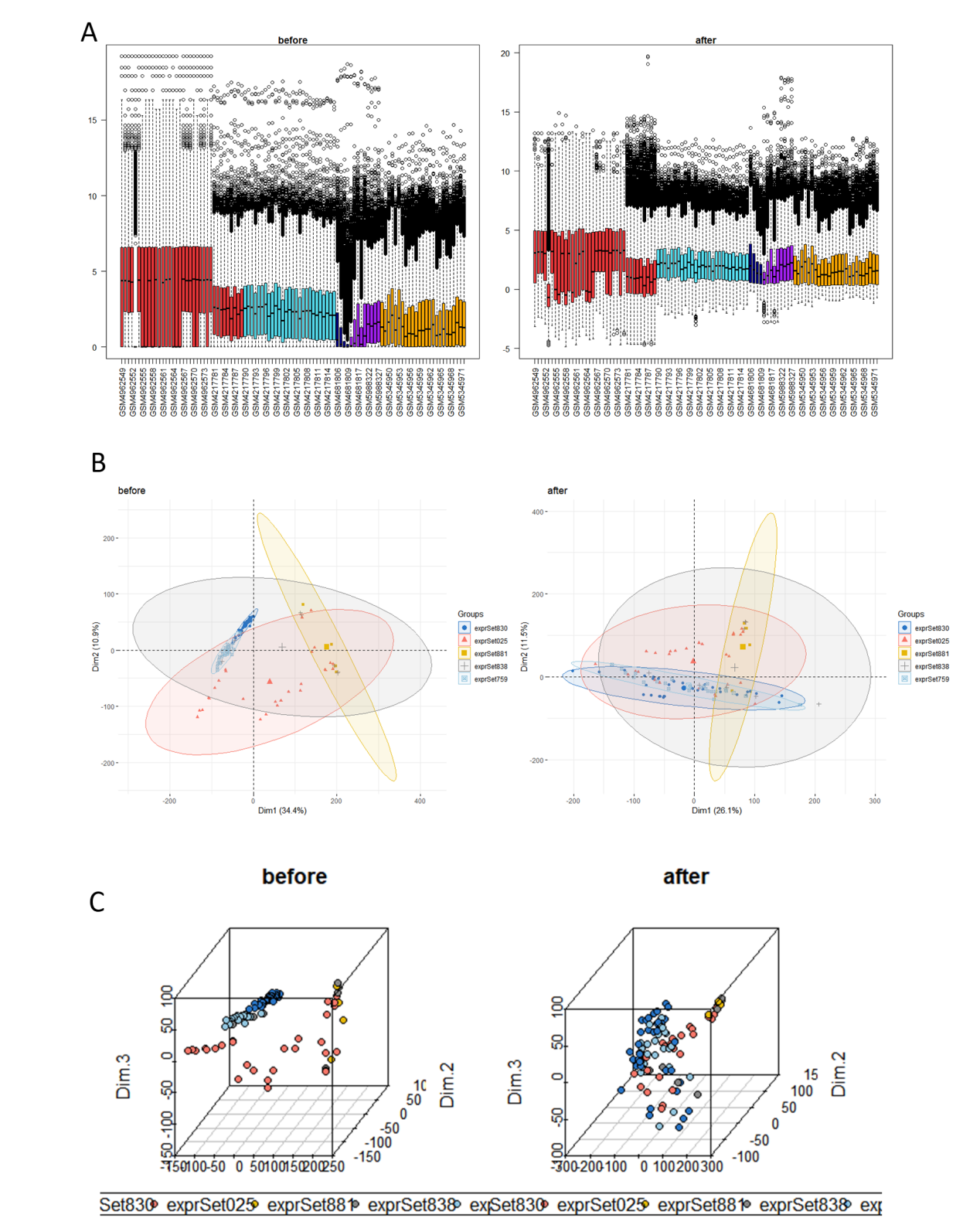


Supplementary figure 2. Data preprocessing. (A, B) PCA of datasets before and after data processing. (C, D) Box plots of datasets before and after data processing. (E, F) 3D projection plots of datasets before and after data processing.


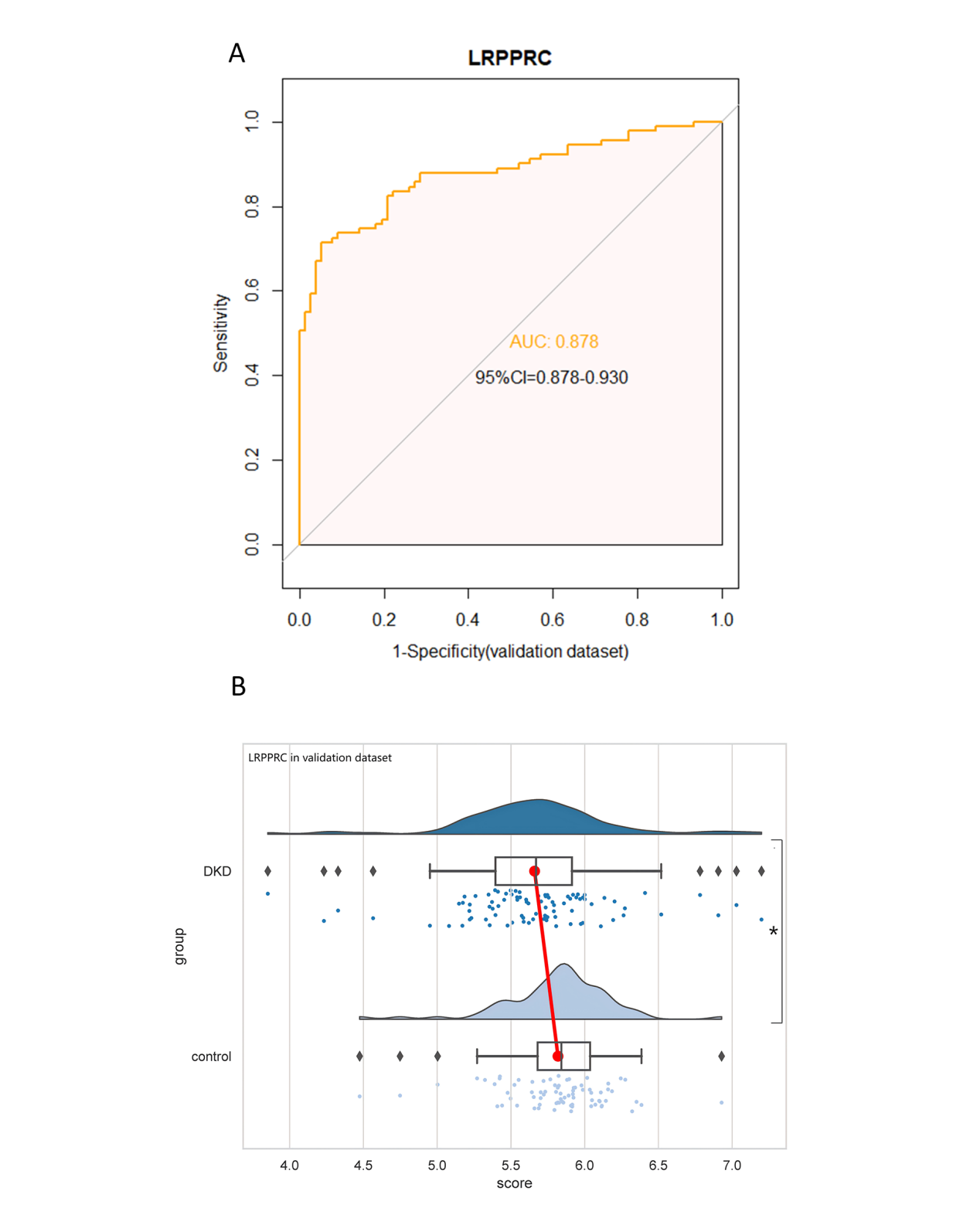


Supplementary figure 3. Expression of LRPPRC in validation dataset. (A) ROC curve for predictive efficacy. (B) Quantitative expression of LRPPRC in DKD.


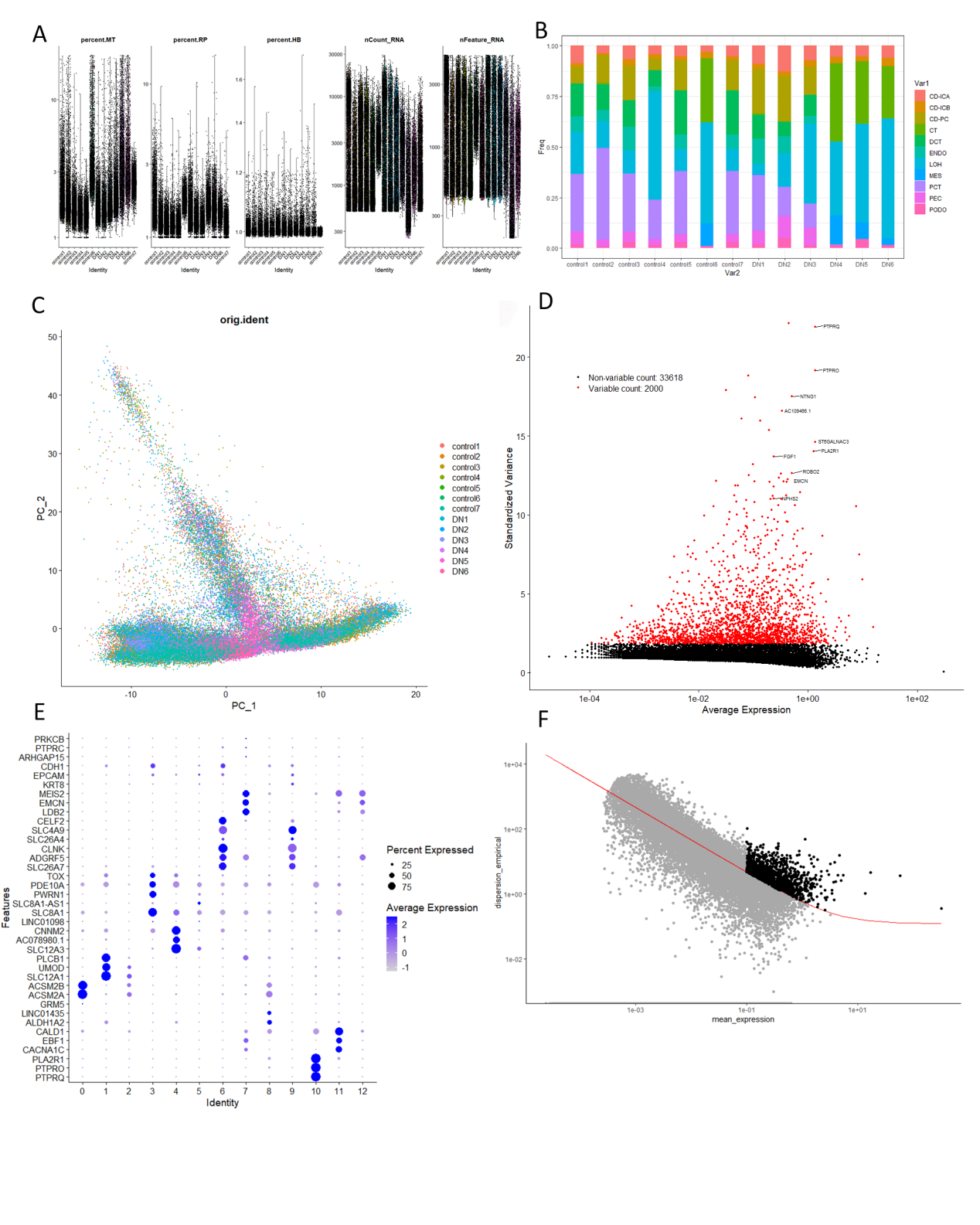


Supplementary figure 4. The quality control and annotation results of the DKD single cell transcriptome data. (A) Quality control in defining the ratio of mitochondrial, ribosome and hemoglobin genes. (B) The cell types between the samples. Cell annotation: mesenchymal cell(MES), glomerular parietal epithelial cell(PEC), proximal convoluted tubular cell(PCT), Loop of Henle cell(LOH), distal convoluted tubular cell(DCT), convoluted tubular cell(CT), collecting duct-principal cell(CD-PC), collecting duct-intercalated cell type A(CD-ICA), collecting duct-intercalated cell type B(CD-ICB), podocyte(PODO) and endothelia cell(ENDO). (C) The elimination of batch effect in the DN (6 samples) and control groups (7 samples, healthy donater). (D) The highly variable genes for dimensional reduction. (E) The correlation between cell markers and clusters. (F)The highly variable genes for pseudotime analysis.
